# Intelligent Beetle Antennae Search for UAV Sensing and Avoidance of Obstacles

**DOI:** 10.3390/s19081758

**Published:** 2019-04-12

**Authors:** Qing Wu, Xudong Shen, Yuanzhe Jin, Zeyu Chen, Shuai Li, Ameer Hamza Khan, Dechao Chen

**Affiliations:** 1 School of Computer Science and Technology, Hangzhou Dianzi University, Hangzhou 310018, China; wuqing@hdu.edu.cn (Q.W.); 172050050@hdu.edu.cn (X.S.); zeno181050017@hdu.edu.cn (Z.C.); 2 Department of Information Science and Electronic Engineering, Zhejiang University, Hangzhou 310018, China; 3150100835@zju.edu.cn; 3 Department of Computing, The Hong Kong Polytechnic University, Hung Hom, Kowloon, Hong Kong 999077, China; shuaili@polyu.edu.hk (S.L.); ameer.h.khan@connect.polyu.hk (A.H.K.)

**Keywords:** UAVs, path planning, obstacle avoidance, MTS, optimization algorithms

## Abstract

Based on a bio-heuristic algorithm, this paper proposes a novel path planner called obstacle avoidance beetle antennae search (OABAS) algorithm, which is applied to the global path planning of unmanned aerial vehicles (UAVs). Compared with the previous bio-heuristic algorithms, the algorithm proposed in this paper has advantages of a wide search range and breakneck search speed, which resolves the contradictory requirements of the high computational complexity of the bio-heuristic algorithm and real-time path planning of UAVs. Besides, the constraints used by the proposed algorithm satisfy various characteristics of the path, such as shorter path length, maximum allowed turning angle, and obstacle avoidance. Ignoring the *z*-axis optimization by combining with the minimum threat surface (MTS), the resultant path meets the requirements of efficiency and safety. The effectiveness of the algorithm is substantiated by applying the proposed path planning algorithm on the UAVs. Moreover, comparisons with other existing algorithms further demonstrate the superiority of the proposed OABAS algorithm.

## 1. Introduction

Unmanned aerial vehicles (UAVs) are increasingly being used in military and civilian environments to perform critical tasks [1,2] because of their ability to complete missions under dangerous conditions or extreme weather [3]. UAVs are often better suited to perform dangerous missions and require real-time updates to avoid drones being discovered or crashed, so we chose drones as our experimental subjects. Computationally efficient planning that generates high quality paths is important for UAVs, which are time-varying nonlinear dynamic systems [4]. Since the moving aircraft and vehicles have high-speed dynamics [5,6], it is an essential requirement to meet the low complexity of planning [7], i.e., to plan the required path in a short time.

UAV path planning can be divided into five types: sampling methods, node-based, mathematical models, bio-heuristic algorithms and multi-fusion algorithms. Sampling based methods include probabilistic roadmaps (PRM) [8], Voronoi maps [9], corridor map [10] and artificial potential field (APF) [11]. These types of algorithms usually need to pre-process the map, grid or sample the map, and then randomly search for paths. These types of path planning algorithms have a time complexity of O(nlogn) to $O(n^{2})$, which is reasonably fast and can be applied to real-time path planning. Node-based path planning algorithms methods include Dijkstra [12], A* [8], lifelong planning algorithm (LPA) [13], harmony search [14], and theta star [15]. These types of path planning have a time complexity between O(logn) and $O(n^{2})$. Due to the low time complexity of these algorithms, real-time path planning is possible. Path planning algorithms based on mathematical models have mixed integer algorithms [16], binary linear algorithm [17] and nonlinear algorithm [18]. The time complexity of such algorithms is described by a polynomial equation and are generally time-consuming, therefore most of these types of algorithms are used in offline planning. The fourth category is bio-heuristic algorithms, including neural networks (NN) [19], genetic algorithm (GA) [20], particle swarm optimization (PSO) [21], ant colony optimization (ACO) algorithm [22] and hybrid leapfrog optimization [23]. These types of path planning have a time complexity of $O(n^{2})$, which can only be applied in static environments for offline path planning. The last category is multi-fusion based algorithms, including PRM node based on optimal algorithm [8], geography informations system (GIS) based 3D path planning algorithm [24], visibility node based on optimal algorithm [25] and 3D path planning robust algorithm [26]. Their time complexity is greater than O(nlogn). Their low time complexity makes them applicable to online path planning.

The heuristic algorithm can solve the NP-hard problem that traditional linear programming can’t solve. We found that heuristic algorithms are often applied to static path planning. Because heuristic algorithms take a long time, it is difficult to cope with real-time dynamic path planning scenarios. Therefore, a bio-heuristic algorithm with fast computation speed and low computational complexity has positive significance for solving the path planning problem of UAV.

### 1.1. Related Work

The path planning of the UAVs needs to meet multiple constraints in a complex environment, and the purpose of these constraints is to enable UAVs to find short and safe paths efficiently. Bio-heuristic algorithms are the methods inspired from biological organisms to solve a problem. People have summarized the behavioral characteristics of organisms that have been continuously evolved by natural selection, so this type of algorithms are efficient.

At present, the primary investment sources and applications of UAVs are in the field of national defense, but they also have great potential for development in the civilian sector. Kroumov et al. [27] proposed a novel potential field based 3D path planning technique for differential drive mobile robots, moving in known environment. Passino [28] provides a tutorial on biology of bacterial foraging optimization (BFO), including an overview of the biology of bacterial foraging. Yang et al. [29] propose a new metaheuristic method, the bat algorithm (BA), based on the echolocation behaviour of bats. These algorithms have strong search performance but have the downside of large time-consumption. Li et al. [30] proposed distributed recurrent neural networks for cooperative control of manipulators. Gawel et al. [31] use UAVs to perform aerial data mapping on heterogeneous grounds using point clouds. In recent years, more and more researches have begun to use bio-heuristic algorithms for path planning of UAVs. This kind of planning method does not need to build a complex environment model but provides a very effective way for path optimization. Researchers have explored the path planning of UAVs in 2D and 3D space. Xu et al. proposed the application of chaotic artificial bee colony (ABC) methods to path planning [32], Duan et al. proposed the application of the hybrid ACO approach to path planning in 3D space [33]. Duan et al. [34] proposed the application of max-min adaptive ACO to the path planning of multiple UAVs. Mittal et al. [35] proposed an offline path planning method based on a multi-objective evolutionary algorithm. Roberge et al. [36] compared the performance of UAV path planning implemented by PSO and GA.

Our algorithm works similarly to PSO and genetic algorithms, so we briefly introduced how they work. The PSO algorithm is group-based and moves individuals in the group to a suitable area based on fitness to the environment. Genetic algorithm is a search algorithm used in computational mathematics to solve optimization. It is a kind of evolutionary algorithm. The intelligent algorithm uses all the path point sets as the optimization target and obtains the best path with the fitness value through the respective search methods.

### 1.2. Organization and Contributions

The structure of this paper is as follows. The problem description is introduced in Section 2, including the definition of path planning and the selection of obstacles. In Section 3, we introduce the methodology, including the path representation, the definition of the cost function and the description of the obstacle avoidance beetle antennae search (OABAS) algorithm. In Section 4, we show the simulation results and compare the performance of this algorithm with other evolutionary algorithms in path planning. In Section 5, we summarize the full paper. Before the end of this section, the main contributions of this article are listed as follows.

- This paper solves the shortest path and obstacle avoidance problem by proposing a novel path planning algorithm, termed OABAS algorithm.
- A linear loss function is designed as a cost function of the OABAS algorithm according to the constraints of UAVs.
- The proposed algorithm is applied to the UAV model and compared with the existing algorithms to verify the feasibility and efficiency.

## 2. Preliminaries

Path planning has a long history of research in robotics. Before the advent of the electric-driven robots, many classic path planning problems, such as the traveling salesman problem (TSP) [37] and the Chinese postman problem [38], have been a focus of academic research. In response to these classic problems, various solutions have been proposed. Since then, along with the emergence of various types of robotic arms [39,40], wheeled robots [41], and humanoid robots [42,43], there is a requirement to study the path planning problems of such devices.

### 2.1. Path Definition and Generation

In this section, we need to present two keywords that are used in robot navigation.

Navigation: the task of navigation is to move a robot from one location to another. In the process of navigation, the robot movement needs a path that is gradually determined. During the navigation, it is often necessary to rely on the sensor data to update the information about position of the surrounding obstacles and to update the position and direction information of the robot.

Path planning: in general, the work that path planning needs to do is to collect relevant data information and generate available paths based on constraints. We use the cost function to constrain the path. The cost function can generally be determined by the length of the path, possibility of collides with obstacles, the load of the robot, and other conditions that cause danger. From this, it can be inferred that reducing the numerical value of the cost function can plan an efficient, feasible and safe path.

According to the working environment with mobile robots, we can divide path planning into two types. One type is the global path planning of the model in a state where the environmental conditions are all known, and can also be called offline path planning. The other is local path planning, also known as online path planning. Local path planning uses the concept of navigation, mentioned above, to update the information about surrounding environment based on the sensor data and perform path planning in real-time to reach a specified destination.

We combine the concept of minimum threat surface (MTS) to ignore the optimization of the flight height value which means that the altitude value of the aircraft is determined by the environment and threat information and the tasks performed by the aircraft. This operation reduces the range of optimization required, and the generation time of the path is significantly reduced.

We specify the starting point and the target point of the UAV and the path planning is performed according to the specified points. This method is described in detail in Section 3, which generates whole path at a time, then uses the proposed OABAS algorithm to adjust the points of the path until these points meet the planning requirements of the path.

### 2.2. Obstacle Sensing and Avoidance in Path Planning

In the global path planning, we can obtain some static target information in the environment, such as mountains and air defense threats. In order to not lose the generality, we used many different types of obstacle environments. We used obstacle-free, a single regular obstacle, multiple regular obstacles, a single irregular obstacle, multiple irregular obstacles and mixed obstacles (including multiple regular and irregular obstacles) for simulations. Such an obstacle environment setting can effectively prevent the algorithm from being useful only in a specific obstacle environment. Since the algorithm can reflect whether the obstacle is successfully avoided (there is a corresponding penalty value to record if it crosses the obstacle successfully), we can count the success rate and compare it with other path planning algorithms. In Section 4.1, we used a bit representation method to represent the presence of a virtual obstacle. In Section 4.4, we used a static cylindrical range to represent the air defense threat in space. In dynamic obstacles, such as abnormal weather areas and flocks, because of their shape uncertainty, we create a minimum enveloping cylinder to represent their range of influence. The moving direction of these dynamic obstacles is to simulate the movement of birds and unusual weather in nature to move.

## 3. Methodology

**Figure 1:**
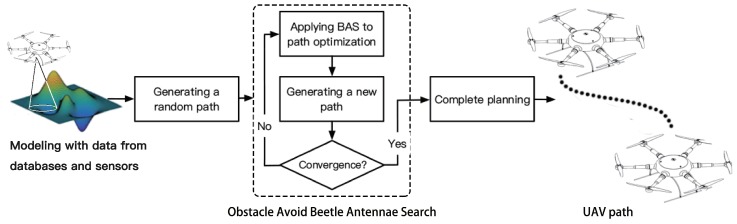
Flowchart of obstacle avoidance beetle antennae search (OABAS) algorithm applied to path planning of unmanned aerial vehicles (UAVs).

Now, we will present a method for path planning in a known environment as shown in Figure 1. First, we read the map information and generated a random initial path, then used this path as input to the OABAS algorithm which adjusted the waypoints until the path met the convergence condition. Finally, we output the qualified path to the UAV for execution. The core of this method is the formulation of the cost function and the optimization of the fitness value by OABAS algorithm. The following content is divided into three parts to introduce this method. Section 3.1 introduces the path generation and the acquisition of environmental information, Section 3.2 explains the structure and meaning of the cost function in detail, and Section 3.3 introduces the proposed OABAS algorithm.

### 3.1. Path Representation

Search techniques (e.g., simulated annealing [44], A* [45]) are generally used to find the optimal solution for a particular function, but large-area path planning in a high-dimensional space is a typical large-scale optimization problem. With the expansion of the search space, the computational cost of searching for the best path in 3D space exponentially increases, therefore this problem is usually called a non-deterministic polynomial-time (NP)-hard problem. When the drone is performing a mission, we can assume that the aircraft maintains a minimum safety clearance with the land plane. The literature [46,47] proposes the concept of the MTS during the movement of the drone, indicating that flying on this surface will have the property that the terrain threat is minimal and the path of the drone is on this surface. This assumption transforms the original 3D path planning problem to a 2D path planning on the MTS. Therefore, in the path planning and path recording of the drone, it is not necessary to record the altitude information of the aircraft. In other words, the altitude information of the aircraft does not need to be involved and recorded in the coding process of the path planning.

(1) $$F\left(x,y\right)=h_{u}+f\left(x,y\right)+Q\left(x,y\right).$$

Equation (1) enables the UAV to have a minimal threat surface so that it can avoid obstacles in the vertical direction. Note that F(x,y) is the height of the aircraft, f(x,y) is the terrain profile, and $h_{u}$ is the specified terrain clearance. The distance $h_{u}$ between the ground and the aircraft can be a special function of the downrange. Also, the obstacles can be defined as Q(x,y). As expressed in Equation (1), the minimum threat surface generated by MTS takes into account terrain, threats, and obstacles. If the ground suddenly rises, the F(x,y) value at this position becomes very large. So according to the concept that our aircraft only fly on the smallest threat surface, we are not inclined to cross large obstacles. From the concept of the MTS mentioned above, the path we need to plan must be on this surface. Since we fixed the starting point and target points of the flight path and connect the path points of each step, the resulting path is also a curve. By projecting the curve onto the MTS according to the corresponding relationship of the projection, the track corresponding to the optimal path on the MTS can be easily obtained. According to the references [48,49,50], we define the drone path planning here. In this paper, we assume that the UAV’s flight path is projected onto a 2D plane, making this space as workspace $U\in R^{k\times k}$ (*k* is the size of map). There are often obstacles in the workspace, so we need to define them. We defined obstacles as O⊂U, and the space of U that is free of obstacles is represented as $U_{\mathrm{fre}}=U\setminus O$. The starting point is $x_{\mathrm{sta}}\in U_{\mathrm{fre}}$, and the target point is $x_{\mathrm{end}}\in U_{\mathrm{fre}}$. We define the path [51] as a vector $\vec{\gamma }\in R^{s}$ (*s* is the number of path points) with the following definitions.

**Definition** **1.** *Path plan: initialize a path $\vec{\gamma }$, where $\gamma_{1}=x_{sta}$ and $\gamma_{s}=x_{end}$. If there is a process $\phi (\cdot ):U\to R^{s}$ that satisfies $\vec{\gamma }\left(\tau \right)\in U_{fre}$, for all τ∈1,2,…,s, then ϕ is called path planning.*

**Definition** **2.** *Path optimization: We give an initialization path $\vec{\gamma }$. Under the constraint of cost function Equation (2), if we satisfy the Definition 1, we find a path ${\vec{\gamma }}^{\prime }$, and $f\left(\gamma^{\prime }\right)=\min\left\{f\left(\vec{\gamma }\right),\;\vec{\gamma }\in \Gamma =\left\{\gamma^{1},\gamma^{2},\ldots ,\gamma^{m}\right\}\right\},m$ is the number of iterations of path planning, then ${\vec{\gamma }}^{\prime }$ is the optimized path, and $\phi^{\prime }$ is the path planning optimization.*

### 3.2. Cost Function

Since the relationship between the UAVs being detected, destroyed, and the state of the aircraft has not been explicitly studied so far, there is no accepted mathematical expression for the UAV’s path cost function. From the cost function of cruise missiles [52,53,54,55], we make corresponding modifications, and apply the previous research results to the trajectory planning cost function of the aircraft, and then summarize the following constraints. First, the trajectory of the aircraft should as short as possible. This constraint reduces flight time, enhances safety and improves energy efficiency. Additionally, the UAVs usually have a maximum turning angle [4]. The maximum turning angle imposes more performance constraint on the aircraft. Last, the aircraft path cannot be too close to obstacles or known threats, because the close spacing between the aircraft and the obstacles can easily lead to accidental collisions. In accordance with these limitations, we propose an improved aircraft path planning cost function

(2) $$f\left(\vec{\gamma }\right)=\sum_{i=2}^{s}\left(k_{1}Li+k_{2}H_{i}+k_{3}T_{i}\right).$$

In Equation (2), $k_{1}$ represents the penalty coefficient of the distance, $k_{2}$ represents the penalty coefficient of the maximum corner, and $k_{3}$ represents the penalty coefficient of the obstacle collision.

(3) $$L_{i}=\sqrt{x^{2}+{\left(\gamma_{i}-\gamma_{i-1}\right)}^{2}}.$$

Equation (3) denotes the contents of $L_{i}$ which represents the distance cost of the *i*th path point, where *x* represents the displacement of the aircraft moving forward each time step (they are equal between themselves). The values $\gamma_{i}$ and $\gamma_{i-1}$ represent the projected length of the distance between the two path points of the aircraft in the y-axis direction

(4) $$H_{i}=\left\{\begin{array}{cc} \;\;1, & \mathrm{if}\;\;x⩾L_{i}\cos\theta ,\\ \;\;0, & \mathrm{otherwise}, \end{array}\right.$$

where $H_{i}$ is a corner penalty factor. When a certain flight path forms an angle larger than the specified maximum angle θ of the aircraft, this value is activated (set to be unity). We assume that the direction at which the aircraft starts is parallel to the positive *x*-axis direction. This imposes a penalty on path cost if the angle become larger than θ.

(5) $$T_{i}=\left\{\begin{array}{cc} \;\;1, & \mathrm{if}\;\;z_{i}\in O,\\ \;\;0, & \mathrm{otherwise}, \end{array}\right.$$

where $T_{i}$ is an obstacle penalty parameter. This value is activated (set to be unity) when there are obstacles near the path to keep away from the obstacles. The parameter $z_{i}$ is the detection range. Due to the concept of MTS mentioned above, the flight altitude is guaranteed to be optimal, and other threat indices depend directly on the constraints. Also, all node coordinates of the path $\vec{\gamma }$ restrict each other. Therefore, the path planned by the proposed method not only enables the aircraft to reach the destination in a short time under the condition of safely avoiding the obstacle, but also meet the performance index constraint of the aircraft.

### 3.3. OABAS Algorithm

**Figure 2:**
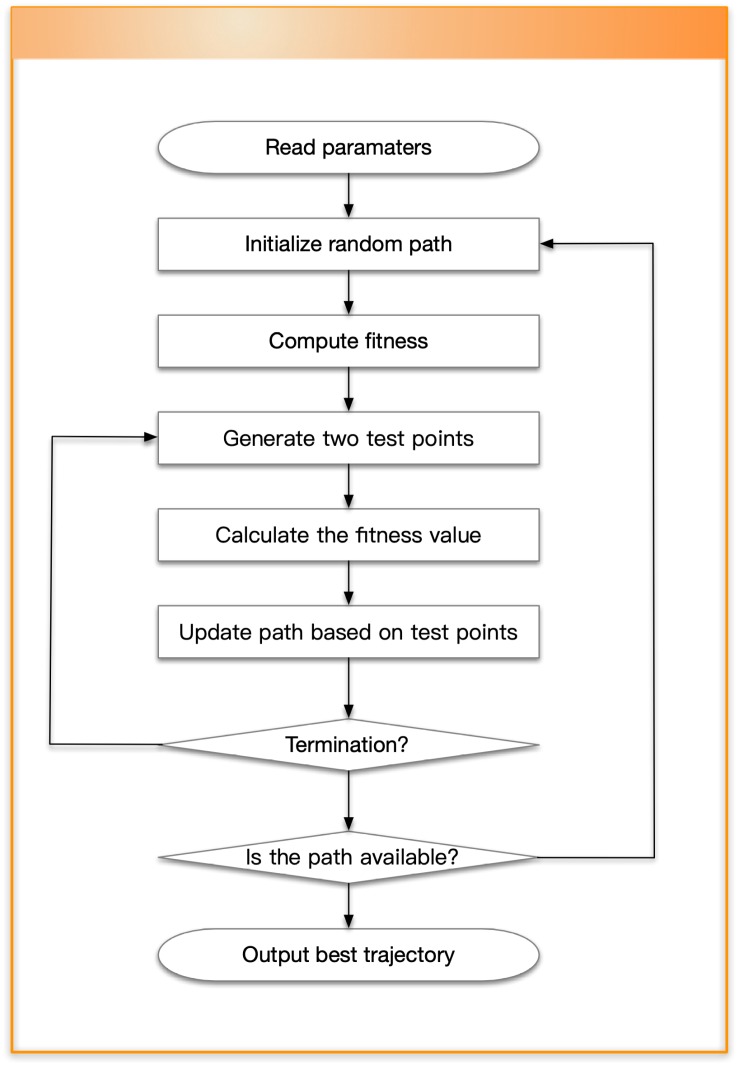
Flowchart of the proposed OABAS algorithm.

Recently, a new metaheuristic strategy called beetle antenna search (BAS) for function optimization was proposed in [56]. After setting the appropriate parameters, this strategy can quickly find the fitness value close to optimal of the objective function. Compared with the bio-heuristic algorithms mentioned in Section 1.1, it has tremendous search speed. We believe that the fast search performance of the BAS strategy fits well with the real-time requirement of the path planning. We improved the path planning algorithm on the basis of BAS and name it OABAS algorithm, and the description of OABAS algorithm is as follows. Figure 2 shows the flow chart of our proposed OABAS algorithm.

First, we get the initial path $\vec{\gamma }$ and calculate the objective fitness value $f(\vec{\gamma })$ to be optimized according to Equation (6).

(6) $$f\left(\vec{\gamma }\right)=\sum_{i=2}^{s}\left(k_{1}\sqrt{x^{2}+{\left(\gamma_{i}-\gamma_{i-1}\right)}^{2}}+k_{2}H_{i}+k_{3}T_{i}\right).$$

The more the path points *s* are used, the higher the accuracy of the planned path, but the path planning will take a long time. Randomly generate the beetle’s search direction $\vec{d}$ and normalize it. In each dimension, the beetle has a corresponding exploration direction. The random direction is

(7) $$\vec{d}=\frac{\mathrm{rands}\left(s,1\right)}{{∥\mathrm{rands}\left(s,1\right)∥}_{2}},$$

where rands $\left(\cdot \right)$ is a function that generates *s*-dimensional random numbers, through which we obtain the normalized direction vector. Then we compare the left and right antenna’s value of the beetle by the following equations

(8) $$\left\{\begin{array}{c} {\vec{\gamma }}_{\mathrm{rig}}^{m}={\vec{\gamma }}^{m-1}+\delta^{m}\vec{d},\\ {\vec{\gamma }}_{\mathrm{lef}}^{m}={\vec{\gamma }}^{m-1}-\delta^{m}\vec{d}, \end{array}\right.$$

where

(9) $$\begin{array}{cc} \delta^{m} & =\zeta^{m}c, \end{array}$$

where $\delta^{m}$ is the length of the beetle’s step size after *m* iterations, and setting this initial value appropriately can make the algorithm have better search performance.

(10) $$\begin{array}{cc} \zeta^{m} & =\zeta^{m-1}\eta , \end{array}$$

where $\zeta^{m}$ is the antennae length after *m* iterations, which is a value that changes as the number of iterations increases. The change of δ is determined by the decay rate η (typically between 0 and 1). Note that *c* is the ratio of the length of the step to the length of the beetle’s antennae. The beetle’s next exploration location is updated by

(11) $${\vec{\gamma }}^{m}={\vec{\gamma }}^{m-1}+\mathrm{sign}\left(f\left({\vec{\gamma }}_{\mathrm{rig}}^{m-1}\right)-f\left({\vec{\gamma }}_{\mathrm{lef}}^{m-1}\right)\right)\delta^{m-1}\vec{d},$$

where $f\left({\vec{\gamma }}_{\mathrm{rig}}^{m-1}\right)\;\mathrm{and}\;f\left({\vec{\gamma }}_{\mathrm{lef}}^{m-1}\right)$ indicate the fitness values of the beetle’s left and right antennae after m−1 iterations, and function sign $\left(\cdot \right)$ is

(12) $$\mathrm{sign}\left(x\right)=\left\{\begin{array}{cc} 1,\; & \mathrm{if}\;x>0,\\ 0,\; & \mathrm{if}\;x=0,\\ -1,\; & \mathrm{otherwise}. \end{array}\right.$$

The algorithm is based on BAS, and its prototype algorithm is improved to be used for UAV path planning.

In order to explain the OABAS algorithm more clearly, we have detailed the steps of the OABAS algorithm in Algorithm 1. The explanation of variables and steps is as follows: $\delta^{0}$ represents the initial exploration step size, and $\zeta^{0}$ represents the initial length of antennae. The optimal fitness value of the cost function is initialized to infinity, and $\gamma^{0}$ represents initial path, which is continuously optimized by OABAS algorithm. When $\vec{\gamma }$ satisfies the convergence condition, the optimized solution ϕ is output and the optimal fitness value $f_{\mathrm{bes}}$ is saved. The remainder of variables are same as as described above.

 **Algorithm 1:** Obstacle avoidance beetle antennae search (OABAS) algorithm for path planning of UAVs.

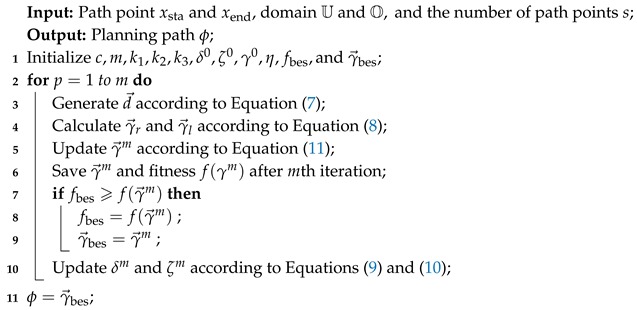



## 4. Simulation Studies and Experimental Results

In this section, we apply the proposed OABAS algorithm to a simulated model of a UAV to test their real-world performance in path planning. In simulations, we use a volumeless and massless particle to simulate UAVs to reduce calculation time. Considering the actual volume of UAVs, we set a minimum safety gap in the algorithm to ensure that UAVs can complete the task of obstacle avoidance. The maps used in the simulation are equal in length and width, and the map information includes only two types of the domain: the feasible domain and the infeasible domain. We use black areas to represent obstacles, and the white area represent free space. In Section 4.1, we apply OABAS algorithm in different types of environments to verify that OABAS algorithm is effective in real-world scenario. In Section 4.2, we set different parameters for the algorithm to obtain the optimal algorithm performance, and also show the statistical effect diagram of the path planning. In Section 4.3, we apply the proposed OABAS algorithm, the conventional ABC and PSO algorithm in same environments and conduct comparative simulations. In Section 4.4, we implemented dynamic obstacles of different sizes and shapes in high-resolution maps by pre-planning future paths, and we assume that these obstacles are only detected when approaching the aircraft.

### 4.1. UAV Path Planning in Different Environments

In this subsection, we apply the OABAS algorithm for obstacle avoidance. Due to the complex environments in the real-world scenarios, whether obstacle avoidance is effective in various types of environments needs to be verified. We set up six types of environments for simulations: obstacle-free, a regular obstacle, multiple regular obstacles, an irregular obstacle, multiple irregular-mixed obstacles.

#### 4.1.1. In Obstacle-Free Environment

This subsection is used to verify that the convergence of the OABAS algorithm are same as in path planning. In this part, we used a map with the length of x=36 and the width of y=36. The starting point $\gamma_{1}$ and the target point $\gamma_{s}$ were set to the leftmost and rightmost centers of the map. The path was a straight line between two points, the theoretical optimal value was $f(\vec{\gamma_{\mathrm{bes}}})=35$. After acquiring the map information, we generated a random path according to the algorithm described in Figure 1.

**Figure 3:**
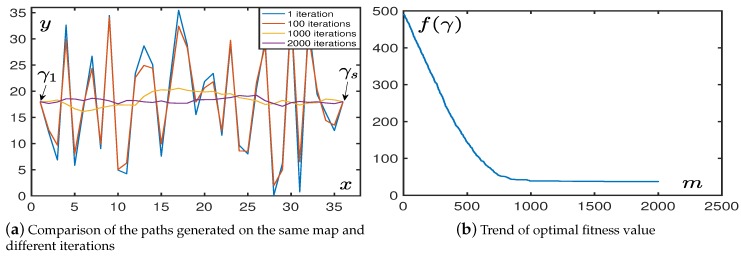
The performance in an environment without obstacles.

According to the curve in Figure 3a, as the number of iterations increased, the generated path had a trend of convergence to the optimal straight line path. We optimized this fitness value to make the UAVs follow the best path under constraint conditions. Figure 3b shows the change in fitness of the planned path.

From the simulation, we find that the OABAS algorithm only takes about 1000 iterations to plan a better path. Based on the results of Table 1, convergence takes time around 0.01 s. Table 1 is a statistical result for UAV path planning in an environment without obstacles. We set the step size δ to be a variable, and 100 simulations are performed using the method of controlling variables. Under different step size δ, we set the decay rate η=0.99995. Then we count the average convergence steps $m_{\mathrm{ave}}$, the average convergence time *t*, average fitness value $f_{\mathrm{ave}}$ after 100 convergences. Table 1 shows the best fitness value $f_{\mathrm{bes}}$ obtained in 100 tests, the standard deviation $f_{\mathrm{Std}}$ of the batch test, and finally we analyze the result and determine if the step is convergence to a reasonable value. The convergence criterion we use here is $f_{\mathrm{ave}}⩽50$, and if the condition is true, we conclude it to be a reasonable step size.

#### 4.1.2. In Regular Obstacles Environment

In this subsection, we test the OABAS algorithm for environments with a single regular obstacle and multiple regular obstacles.

**Figure 4:**
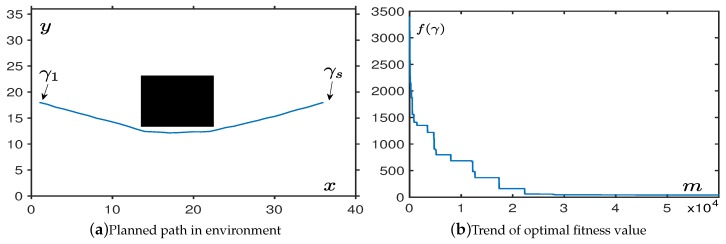
The performance in an environment with a regular obstacle.

As shown in Figure 4a, a black square in the center area is used to represent an obstacle. In order to the visually observe the effect of obstacle avoidance, we set the connection points between the starting point and the target, to pass point through the obstacle area. We can clearly understand that the path we finally planned is successfully avoiding obstacles, and there is a certain safety distance between the path and the obstacle to avoid collisions.

Figure 4b shows the number of iterations required for the algorithm to converge. It was more than the obstacle-free environment in Section 4.1.1, which was roughly around $2.2\times 10^{4}$ iterations. Later, we performed a more detailed performance test at Table 2 to show the relationship between step size δ and OABAS algorithm performance in the environment with a regular obstacle. The specific indicators were the same as those used in Section 4.1.1. In Table 2, we can see that the convergence in the environment with a single obstacle was very fast, but a too small initial step size will cause the algorithm to fall into the optimum local value prematurely. The convergence criterion we used here was $f_{\mathrm{ave}}⩽100$, and if the condition was true, we concluded it to be a reasonable step size. The reason for algorithm falling into a local optimal value was that the $f_{\mathrm{ave}}$ value was large, proving that most of the paths were not successfully planned.

**Figure 5:**
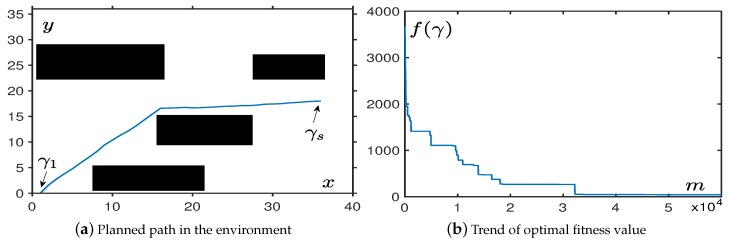
The performance in the environment with multiple regular obstacles.

As shown in Figure 5a, we had set up a number of rectangular obstacles of different specifications. In order to achieve the visual effect of obstacle avoidance, we set the connection points of the starting point and the target point, to pass through several obstacle areas.

From Figure 5a, we can clearly understand that the path we finally planned is successfully avoiding obstacles. Figure 5b shows that the algorithm converged at roughly the same speed as the performance in an environment with a regular obstacle. Later, we performed a more detailed performance test as shown in Table 3, and we calculated the effect of different step sizes on the performance of OABAS algorithm in the case of multiple regular obstacles. As shown in Table 3, the OABAS algorithm converged very quickly with a reasonable step size δ in the environment with multiple obstacles, but an excessive initial step size δ made it challenging to converge to a better value later. Therefore, the step size of the algorithm should be set within a reasonable range to avoid setting a huge initial step size for a wide range of searches. The convergence criterion we use here was $f_{\mathrm{ave}}⩽100$, and if the condition was true, we concluded this it to be a reasonable step size.

#### 4.1.3. In Irregular Obstacle Environments

As we have verified, OABAS algorithm has good simulation results in the environment without obstacles and in the environment with regular obstacles. In this subsection we simulation with the environment with irregular obstacles.

**Figure 6:**
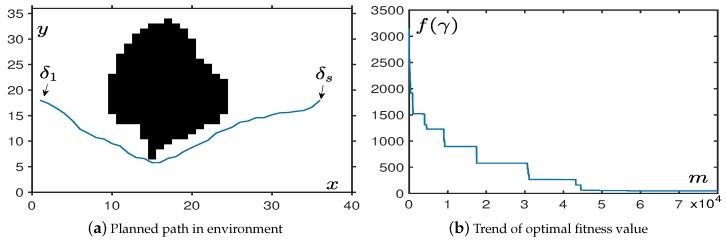
The performance in an environment with an irregular obstacle.

In the first simulation, we add a large irregular obstacle to test more comprehensive performance. We use black areas to represent obstacles that are continuous in the center of the map. In order to visually observe the effect of obstacle avoidance, we set the starting point $\gamma_{1}$ and the target point $\gamma_{s}$ such that the straight line between them passes through the obstacles. It can be seen from Figure 6, OABAS algorithm can accomplish the requirements of the short path, avoiding obstacles and maintaining safe separation from obstacles. Figure 6 shows that when we plan the path in an environment with an irregular obstacle, the number of iterations for convergence is about twice that of the previous simulations. In this environment, we find from Table 4 that it is different from the previous environment. At the same time, the failure rate in this environment has also increased significantly, so the convergence criteria is appropriately adjusted to $f_{\mathrm{ave}}⩽150$.

**Figure 7:**
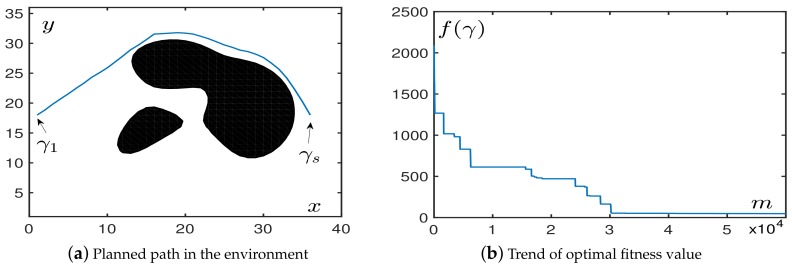
The performance in an environment with multiple irregular obstacles.

In the second simulation, we generated multiple irregular obstacles located in the middle and back of the map. It can be seen from Figure 7a that the algorithm can complete the task of obstacle avoidance with an environment with multiple irregular obstacles. Figure 7b shows that the time spent was roughly the same as spent in the environment with a single irregular obstacle. In this simulation, we find from Table 5 that irregular obstacles needed more iterations to converge, but had a higher success rate. We still set the convergence criterion in this environment to be $f_{\mathrm{ave}}⩽100$.

#### 4.1.4. In Mixed Obstacle Environments

We placed both regular and irregular obstacles in the map to test the performance of OABAS algorithm. As with the previous environment, it was necessary to avoid multiple obstacles in order to reach the endpoint located in the midpoint on the right from the starting point in the lower left corner.

**Figure 8:**
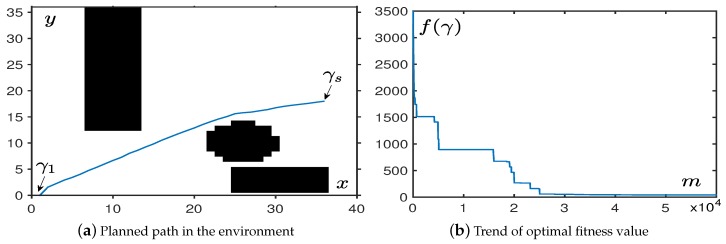
The performance in an environment with mixed types of obstacles.

Figure 8a shows that OABAS successfully avoids obstacles and path length was short with short part length. Figure 8b shows that in the convergence speed of the algorithm was roughly the same as the environment with regular obstacles and obstacle-free.

Finally, we performed a more detailed performance test as shown in Table 6, which quantifies the performance impact of different step sizes on OABAS algorithm in the case of mixed obstacles. The OABAS algorithm converged very quickly in the environment with mixed obstacles, and the step size δ should be set within a reasonable range for a wide range of searches.

From the above simulations, we can observe that the performance of the OABAS algorithm changes in different types of environments. These simulations demonstrate that OABAS algorithm performs well in different types of environments. Although there are some differences in the performance of the OABAS algorithm in different types of environments, most of the planned paths have a high success rate and can converge quickly (visible in Section 4.1.3).

### 4.2. Cycle Batch Tests

The content of this section explores the impact of different step size δ and step attenuation rate η on path planning success rates ψ. In order to find parameters that can generate as many successful paths as possible, we performed multiple tests with different δ and η. In this experiment, we set the number of iterations to 1000. A smaller number of iterations resulted in a more significant difference in success rates, making it easier to select the optimal set of step and attenuation rate parameter pairs. But this was not our actual planning success rate. The sample size of the simulation was 100. Success rate ψ was calculated after OABAS algorithm generated 100 paths in the simulation of multiple irregular obstacles. Finally, the optimal δ and η were obtained, and the targeted recurrence test was carried out. That is, the simulation performed several times with the same value of δ and η which generated a thousand paths and was used to represent the quality of paths visually.

The Table 7 is the result of the path generated by the combination of different parameters. The horizontal axis represents the initial δ of 1 to 9, and the vertical axis represents η of 0.99991 to 1 (a total of ten levels). The values in the table are the number of successful paths generated after 100 trials.

**Figure 9:**
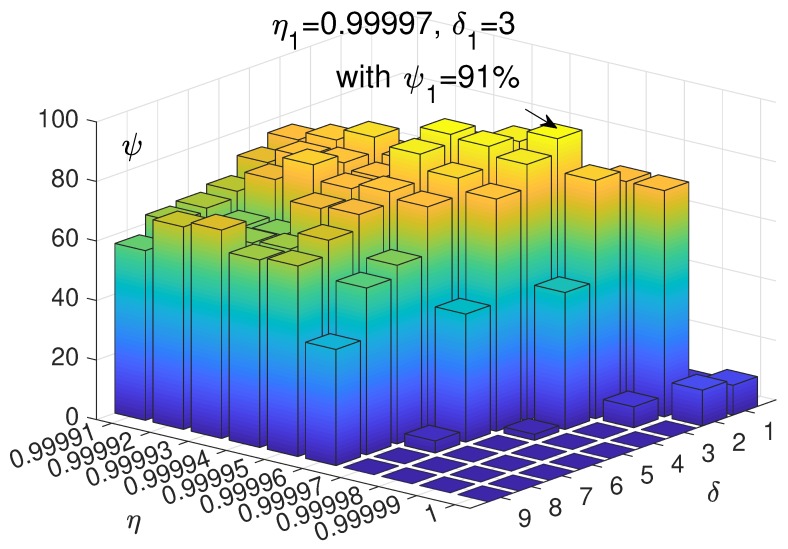
Effect of step and decay rate on success rate of OABAS algorithm.

We visualize it as shown in Figure 9 to show its distribution. In this histogram, the effect of the two parameters δ and η on the convergence effect is more obvious. The better results are concentrated in, the more appropriate δ and η.

**Figure 10:**
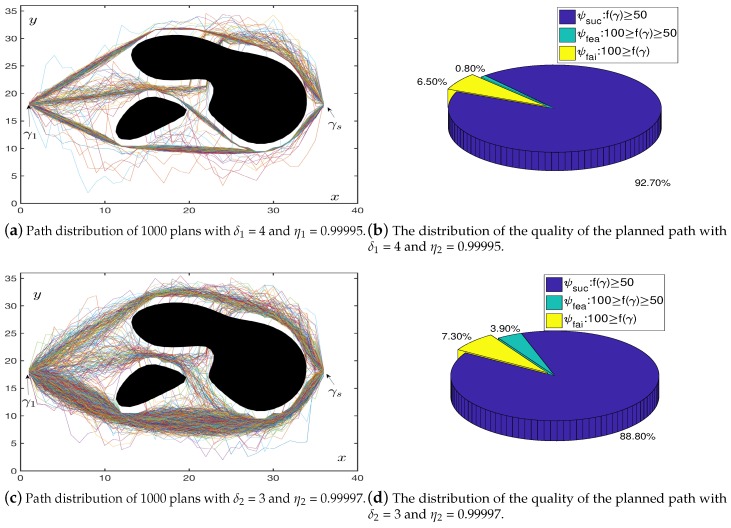
Path distribution in the case of fixed step size and decay rate.

In Figure 10b,d, we used the blue part $\psi_{\mathrm{suc}}$ to represent the number of excellent paths generated. The green part $\psi_{\mathrm{fea}}$ is used to indicate the number of available paths generated which means this types of the path with a longer path length, but satisfying the maximum turning angle and avoiding the obstacles. Finally, we used the yellow part $\psi_{\mathrm{fai}}$ to indicate the number of failed paths generated. According to the above statistical simulation, when $\delta_{1}=4$ and $\eta_{1}=0.99995$, the path planning had a success rate $\psi_{1}=93.7\%$. In addition, when $\delta_{2}=3$ and $\eta_{2}=0.99997$, there was also a high success rate $\psi_{2}=92.7\%$. The convergence properties of the OABAS algorithm were similar to those of the BAS on the objective function, and both the appropriate δ and η were needed to achieve an optimal convergence.

### 4.3. Comparisons with PSO and ABC

In order to compare the performance between the OABAS algorithm and traditional bio-heuristic algorithms in the path planning, we need to make a more detailed evaluation. Unlike Section 4.2, after obtaining the best parameters, our OABAS uses an iteration step of 50000 to ensure the reliability of path planning.

We performed multiple simulations and took the average value to eliminate contingency and ensure that the results were indicative of real scenarios. We wanted to get the path length as short as possible. At the same time, the path planning success rate was also one of the evaluation criteria for evaluating whether a path planning algorithm has good quality or not. The path planning algorithm that does not have a reliable and stable result will lead to very serious consequences when the UAVs are flying in the real world. Therefore, the higher the success rate of path planning, the better. So we counted the following indicators of the algorithm in Table 8 and Table 9, the average convergence time *t* of the algorithm, the average convergence fitness value $f_{\mathrm{ave}}$ of the algorithm and the success rate ψ of the algorithm.

On the map of size *k*, we set the number of path points $s_{1}=k/2$ and $s_{2}=k$. First, we set the path point to $s_{1}$. In this case, a show from the Table 8 that the OABAS algorithm leads the time indicator. However, after the visual observation of the paths, we found that the UAVs will collide with obstacles. After analysis, we believe that too few path point settings will lead to rough path planning so that UAV will collide with obstacles.

After checking the path, fewer nodes were found to cause the flight path to avoid obstacles, so we needed to set up more nodes for path planning. More nodes will lead to an increase in planning time. We set the number of path points to $s_{2}$ and tested again in the same situation. In the visualization results, the path was successfully planned, in this case did not collide with the obstacle, which proves that the number of path points we selected was reasonable. In Table 9, the OABAS algorithm was faster than other algorithms. The OABAS algorithm achieved a very high success rate in each case while the PSO was challenging to converge due to too many path points. Under the premise of guaranteeing the success rate, the efficiency of the OABAS algorithm was much better, and the time complexity of the ABC algorithm was high.

### 4.4. Tested Results on High-Resolution Maps with Various Types of Obstacles

**Figure 11:**
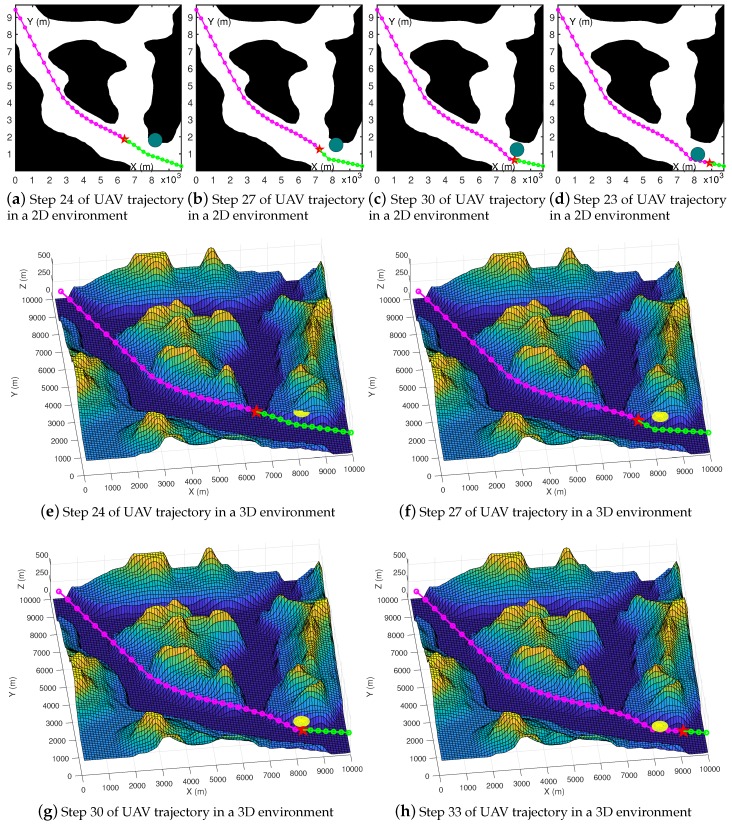
2D and 3D visualization of dynamic obstacle avoidance processes in the virtual map.

**Figure 12:**
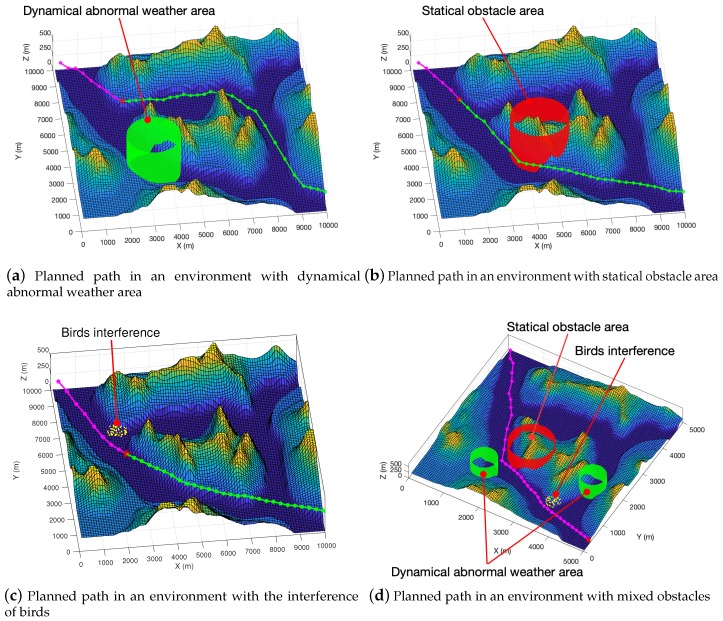
3D visualization of small size (with diameter being 35 m) UAV with various types of obstacle avoidance in the virtual map.

**Figure 13:**
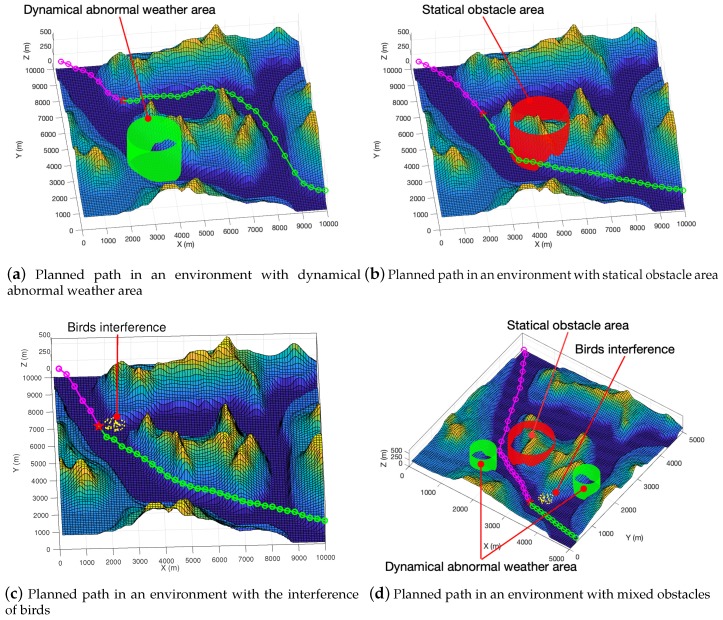
3D visualization of a large (with diameter being 100 m) UAV with various types of obstacle avoidance in the virtual map.

We used more high-resolution test maps from the GeoBase repository [57] and ran experiments in a dynamic environment. By pre-planning the unpassed path, we implement a simple dynamic path planning function. We assumed that there was a dynamic obstacle in the environment, and used the dark green circle in the picture to represents it. We set the UAV’s sensing range 800 m in the real environment, and obstacles within 800 m of distance from the UAV were be discovered. In the experiment, the size of the UAV was generally small, so we did not consider its specific shape and model it according to its specific size. We took the larger of the fuselage length and the length of the wing as diameter to draw hollow circles to represent the UAV. For example, the Global Hawk has a length of 13.5 m and a wing length of 35 m, so we chose 35 meters as the model’s parameters. In the experiment, we used 50 m as the diameter to draw hollow circles for modeling in Figure 11 and Figure 12, and used 100 m as the diameter of the UAV in Figure 13.

A red star indicates where the UAV is currently located. Green circles and line segments represent pre-planned paths. The dark green dot represents an obstacle in the 2D environment that exists in the minimum threat surface of the UAV and poses a threat to the path. In the iterative process, dynamic path planning can try to plan a better pre-planning path while avoiding obstacles.

In Figure 11, the magenta circles and line segments indicate where the UAV has passed, and they will not change. Figure 11a–d show the planning effect of the algorithm in 2D space, and experimental results show that the planned path can avoid obstacles and has a short total path length. We mapped the planned paths in the 2D environment to the 3D environment. Figure 11e–h show our experimental results for dynamic obstacles avoidance in high-resolution 3D maps. The golden sphere represents an obstacle in the 3D environment that exists in the minimum threat surface of the UAV.

From the experiment, we know that OABAS can perform dynamic obstacle avoidance and path planning tasks in high-resolution maps. In the experiment, the time required for each re-planning took less than one second. For example, the MiG-25 is one of the fastest flying fighters in the world [58]. It has a maximum flying speed of up to 1200 km/h at the sea level distance and can fly 333 m in one second. The OABAS algorithm can quickly generate a longer track during one second, so there is plenty of time to calculate the trajectory during the flight.

In general, surface-to-air missiles are mostly guided by radar or radar and infrared. Since the missile poses a significant threat to the aircraft, we assume that the radar’s detection range is a static cylindrical area whose radius is the radar’s detection range. In addition to this, birds often pose a potential threat to UAV in the air. When the airborne radar can detect nearby birds, we establish a minimum circle according to its distribution [59] and consider it as a single obstacle. Building a regular model for an irregular obstacle simplifies the calculation process while avoiding the algorithm spending too much time building the model. The flying speed of birds is between 40 and 80 km/h, and our experiment set it to 80 km/h. In addition to ground threats and birds, there are also unusual weather areas in space. Due to the complexity of the weather, we use a green cylindrical area to indicate that there is unusual weather in the area. Referring to the general typhoon moving speed, we set the moving speed to 36 km/h.

In Figure 12a, we use the green cylinder area to represent the abnormal weather area. We plan an area of abnormal weather to move at a speed of 36 km/h, and the moving direction was random. From the graphical results, our path planning algorithm can effectively plan the path to avoid the abnormal weather area. In Figure 12b, we use a red cylinder to represent a static obstacle, such as surface-to-air missile threats and radar detection from the ground. As can be seen from the Figure 12b, our planned path can avoid dangerous areas and plan a good path. In Figure 12c, we use yellow scatter to represent the flock of birds. The speed at which the birds move was set to 80 km/h, and their direction of movement is random. From the experimental results, we can see that our algorithm can effectively avoid the interference of birds. In Figure 12d, we mixed a variety of obstacles for testing. Specifically, it includes the ground threat represented by the red cylinder, the abnormal weather area indicated by the green cylinder, and the bird group represented by the yellow scatter. From the experimental results, we can still get a safe and short path.

In Figure 13, we used a larger size of the aircraft for obstacle avoidance experiments. Hollow circles with a diameter being 100 m was established in the experiment to represent the aircraft model. Even though the model we use was much larger than the real model of an ordinary drone, we can still observe in the experiment that the path planned by the OABAS algorithm can maintain a safe distance from the obstacles.

## 5. Conclusions

This paper has proposed a novel path planning algorithm called OABAS algorithm. Based on the BAS strategy, a new UAVs path planner has been proposed. The constraints used in this algorithm have taken into account the requirements of shorter path lengths, maximum turning angles, and obstacle avoidance. This paper has combined the MTS to plan for efficient and secure requirements. Besides, we have applied this intelligent path planning algorithm to UAVs simulations. Moreover, this paper has verified the universal applicability of OABAS algorithm obstacle avoidance and compared it with two other bio-heuristic algorithms to prove the effectiveness of the algorithm. As a final remark of this paper, to the best of authors’ knowledge, this is the first work in the field of intelligent optimization that can elegantly design an effective and fast path planner by leveraging the proposed OABAS algorithm.

## Figures and Tables

**Table 1:** Relationship between step size and OABAS algorithm performance in an environment without obstacles.

δ	$m_{\mathrm{ave}}$	T(s)	$f_{\mathrm{ave}}$	$f_{\mathrm{bes}}$	$f_{\mathrm{Std}}$	Con.
0.5	10,503	0.029	35.51	35.30	0.010	Yes
**1.0**	**20418**	**0.057**	**35.47**	**35.29**	**0.008**	**Yes**
1.5	23,873	0.067	35.60	35.40	0.010	Yes
2.0	24,077	0.067	35.92	35.54	0.035	Yes
2.5	24,122	0.067	36.40	35.85	0.077	Yes
3.0	23,957	0.067	36.97	36.03	0.105	Yes
3.5	24,128	0.067	37.53	36.31	0.265	Yes
4.0	24,031	0.067	38.24	36.98	0.366	Yes
4.5	24,243	0.068	39.11	37.70	0.816	Yes
5.0	24,103	0.067	40.05	37.50	1.131	Yes
5.5	24,085	0.067	40.76	38.48	1.429	Yes
6.0	24,186	0.067	41.59	39.56	1.145	Yes
6.5	24,115	0.067	42.60	39.36	3.033	Yes
7.0	24,039	0.067	43.74	41.14	1.910	Yes
7.5	24,105	0.067	44.82	41.76	3.693	Yes
8.0	24,141	0.067	46.79	42.03	7.127	Yes
8.5	24,269	0.068	47.21	43.74	4.717	Yes
9.0	24,195	0.067	48.56	44.02	6.020	Yes
9.5	24,155	0.067	49.69	45.13	7.214	Yes
10	24,055	0.067	50.98	45.00	8.315	No

**Table 2:** Relationship between step size and OABAS algorithm performance in the environment with a regular obstacle.

δ	$m_{\mathrm{ave}}$	t(s)	$f_{\mathrm{ave}}$	$f_{\mathrm{bes}}$	$f_{\mathrm{Std}}$	Con.
0.5	6819	0.019	858.32	557.84	$1.30\times 10^{4}$	No
1.0	4532	0.013	620.79	38.25	$6.64\times 10^{4}$	No
1.5	7898	0.022	228.38	37.28	$5.69\times 10^{4}$	No
2.0	12,284	0.035	104.18	37.33	$1.94\times 10^{4}$	No
2.5	17,209	0.049	70.35	37.83	$8.45\times 10^{3}$	Yes
3.0	19,065	0.054	55.54	37.71	$3.21\times 10^{3}$	Yes
3.5	19,984	0.057	58.37	38.61	$3.09\times 10^{3}$	Yes
4.0	21,096	0.060	53.17	39.17	$3.45\times 10^{3}$	Yes
**4.5**	**22,111**	**0.063**	**49.58**	**39.73**	$9.73\times 10^{3}$	**Yes**
5.0	22,851	0.065	53.79	39.80	$3.05\times 10^{3}$	Yes
5.5	22,893	0.065	53.80	41.49	$1.48\times 10^{3}$	Yes
6.0	23,313	0.066	51.11	41.58	$1.62\times 10^{3}$	Yes
6.5	23,143	0.066	68.48	42.31	$4.73\times 10^{3}$	Yes
7.0	23,556	0.067	68.72	42.66	$4.44\times 10^{3}$	Yes
7.5	23,436	0.066	59.53	43.64	$1.87\times 10^{3}$	Yes
8.0	23,476	0.067	75.87	44.90	$3.98\times 10^{3}$	Yes
8.5	23,664	0.067	86.04	45.65	$6.28\times 10^{3}$	Yes
9.0	23,605	0.067	114.23	47.07	$2.35\times 10^{4}$	No
9.5	23,595	0.067	132.36	50.07	$1.28\times 10^{4}$	No
10	23,508	0.067	153.99	51.96	$1.10\times 10^{4}$	No

**Table 3:** Relationship between step size and OABAS algorithm performance in an environment with multiple rule obstacles.

δ	$m_{\mathrm{ave}}$	t(s)	$f_{\mathrm{ave}}$	$f_{\mathrm{bes}}$	$f_{\mathrm{Std}}$	Con.
0.5	4897	0.014	46.04	42.41	$2.99\times 10^{2}$	Yes
1.0	6292	0.018	46.31	42.78	$4.02\times 10^{2}$	Yes
1.5	9382	0.027	51.73	42.83	$6.31\times 10^{2}$	Yes
2.0	12,627	0.036	50.30	42.38	$4.47\times 10^{2}$	Yes
**2.5**	**15,906**	**0.045**	**48.06**	**42.84**	$2.23\times 10^{2}$	**Yes**
3.0	18,766	0.053	51.87	42.77	$8.54\times 10^{2}$	Yes
3.5	20,210	0.057	55.10	43.29	$1.27\times 10^{3}$	Yes
4.0	21,267	0.060	54.54	43.75	$1.04\times 10^{3}$	Yes
4.5	22,036	0.063	62.07	43.74	$2.76\times 10^{3}$	Yes
5.0	22,445	0.064	71.34	44.34	$1.43\times 10^{4}$	Yes
5.5	23,327	0.066	66.52	44.60	$2.65\times 10^{3}$	Yes
6.0	23,063	0.065	93.94	46.28	$2.79\times 10^{4}$	Yes
6.5	23,468	0.067	116.06	46.03	$4.88\times 10^{4}$	No
7.0	22,938	0.065	176.84	47.59	$8.61\times 10^{4}$	No
7.5	23,541	0.067	147.41	49.04	$4.92\times 10^{4}$	No
8.0	23,613	0.067	181.11	47.65	$3.59\times 10^{4}$	No
8.5	23,581	0.067	164.87	50.87	$1.86\times 10^{4}$	No
9.0	23,522	0.067	302.28	54.17	$9.23\times 10^{4}$	No
9.5	23,794	0.067	267.42	54.26	$3.56\times 10^{4}$	No
10	23,399	0.066	393.70	54.07	$1.25\times 10^{5}$	No

**Table 4:** Relationship between step size and OABAS algorithm performance in an environment with a single irregular obstacle.

δ	$m_{\mathrm{ave}}$	t(s)	$f_{\mathrm{ave}}$	$f_{\mathrm{bes}}$	$f_{\mathrm{Std}}$	Con.
0.5	2076	0.006	839.27	440.39	$8.31\times 10^{3}$	No
1.0	4833	0.014	578.47	45.59	$8.40\times 10^{4}$	No
1.5	9304	0.026	235.70	45.79	$8.58\times 10^{4}$	No
2.0	15,680	0.044	185.26	45.01	$6.73\times 10^{4}$	No
**2.5**	**21454**	**0.061**	**117.10**	**45.91**	$3.33\times 10^{4}$	**Yes**
3.0	27,507	0.078	127.29	45.32	$2.67\times 10^{4}$	Yes
3.5	31,483	0.089	136.02	45.31	$3.38\times 10^{4}$	Yes
4.0	35,306	0.100	137.72	45.12	$2.75\times 10^{4}$	Yes
4.5	37,304	0.106	187.19	44.38	$4.92\times 10^{4}$	Yes
5.0	44,019	0.125	136.82	44.42	$2.51\times 10^{4}$	Yes
5.5	47,124	0.134	130.63	45.73	$2.52\times 10^{4}$	Yes
6.0	48,738	0.138	159.98	44.61	$3.49\times 10^{4}$	No
6.5	50,540	0.143	145.77	45.68	$3.29\times 10^{4}$	Yes
7.0	53,528	0.152	134.59	45.33	$2.69\times 10^{4}$	Yes
7.5	55,753	0.158	157.71	45.14	$3.58\times 10^{4}$	No
8.0	58,812	0.167	129.98	45.34	$2.51\times 10^{4}$	Yes
8.5	59,677	0.169	185.15	45.04	$4.78\times 10^{4}$	No
9.0	62,318	0.177	178.01	45.54	$4.70\times 10^{4}$	No
9.5	64,671	0.183	145.06	46.25	$3.24\times 10^{4}$	Yes
10	66,818	0.190	153.18	45.71	$3.79\times 10^{4}$	No

**Table 5:** Relationship between step size and OABAS algorithm performance in an environment with multiple irregular obstacles.

δ	$m_{\mathrm{ave}}$	t(s)	$f_{\mathrm{ave}}$	$f_{\mathrm{bes}}$	$f_{\mathrm{Std}}$	Con.
0.5	17,100	0.049	1131.63	1011.86	$5.84\times 10^{2}$	No
1.0	14,902	0.042	1029.72	667.69	$9.34\times 10^{3}$	No
1.5	8053	0.023	700.90	44.30	$7.98\times 10^{4}$	No
2.0	12,208	0.035	324.22	44.21	$7.84\times 10^{4}$	No
2.5	18,918	0.054	112.02	43.19	$1.64\times 10^{4}$	No
3.0	23,355	0.066	107.40	43.46	$9.18\times 10^{3}$	No
3.5	29,487	0.084	92.42	44.40	$6.57\times 10^{3}$	Yes
**4.0**	**35,404**	**0.100**	**75.76**	**43.80**	$3.76\times 10^{3}$	**Yes**
4.5	38,980	0.111	77.82	43.39	$5.02\times 10^{3}$	Yes
5.0	41,242	0.117	93.62	43.02	$6.82\times 10^{3}$	Yes
5.5	44,172	0.125	83.76	43.41	$5.12\times 10^{3}$	Yes
6.0	48,920	0.139	86.69	43.28	$4.53\times 10^{3}$	Yes
6.5	50,991	0.145	83.37	44.16	$4.41\times 10^{3}$	Yes
7.0	52,113	0.148	82.61	44.49	$3.44\times 10^{3}$	Yes
7.5	57,042	0.162	86.57	44.02	$6.95\times 10^{3}$	Yes
8.0	55,957	0.159	96.58	44.46	$7.09\times 10^{3}$	Yes
8.5	58,915	0.167	79.12	44.29	$3.62\times 10^{3}$	Yes
9.0	61,916	0.176	84.94	42.79	$5.25\times 10^{3}$	Yes
9.5	62,468	0.177	101.00	44.37	$7.51\times 10^{3}$	No
10	66,231	0.188	70.62	44.01	$3.26\times 10^{3}$	Yes

**Table 6:** Relationship between step size and OABAS algorithm performance in an environment with mixed types of obstacles.

δ	$m_{\mathrm{ave}}$	t(s)	$f_{\mathrm{ave}}$	$f_{\mathrm{bes}}$	$f_{\mathrm{Std}}$	Con.
0.5	12,488	0.035	40.53	39.97	0.11	Yes
1.0	17,521	0.050	40.22	39.91	0.08	Yes
1.5	21,086	0.060	40.40	39.99	0.22	Yes
2.0	21,694	0.062	40.95	40.10	2.12	Yes
2.5	22,186	0.063	44.53	40.42	$5.73\times 10^{2}$	Yes
3.0	22,336	0.063	47.37	40.49	$8.52\times 10^{2}$	Yes
**3.5**	**23,226**	**0.066**	**43.37**	**41.07**	$1.24\times 10^{2}$	**Yes**
4.0	23,170	0.066	49.39	41.67	$8.93\times 10^{2}$	Yes
4.5	23,319	0.066	49.12	42.22	$1.26\times 10^{3}$	Yes
5.0	23,189	0.066	58.41	42.75	$2.70\times 10^{3}$	Yes
5.5	23,420	0.066	73.59	43.10	$6.83\times 10^{3}$	Yes
6.0	23,131	0.066	67.96	42.91	$3.91\times 10^{3}$	Yes
6.5	23,575	0.067	86.27	42.75	$3.71\times 10^{4}$	Yes
7.0	23,796	0.067	100.55	44.47	$2.71\times 10^{4}$	No
7.5	23,672	0.067	133.62	45.15	$4.51\times 10^{4}$	No
8.0	23,706	0.067	135.61	46.58	$2.00\times 10^{4}$	No
8.5	23,769	0.067	158.27	47.54	$4.26\times 10^{4}$	No
9.0	23,803	0.068	205.10	50.41	$5.27\times 10^{4}$	No
9.5	23,882	0.068	248.31	50.15	$1.30\times 10^{5}$	No
10	23,799	0.068	395.42	51.71	$1.39\times 10^{5}$	No

**Table 7:** Influence of step size and attenuation rate on the success rate of path planning.

	δ	1	2	3	4	5	6	7	8	9
η										
0.99991		0	35	67	76	72	66	64	63	57
0.99992		0	35	72	79	77	73	61	58	68
0.99993		2	43	73	83	78	81	61	62	70
0.99994		1	51	79	78	76	76	73	63	63
0.99995		1	49	82	**90**	87	78	74	69	64
0.99996		1	55	87	89	82	76	60	56	39
0.99997		1	66	**91**	86	78	43	4	0	0
0.99998		2	76	80	46	2	0	0	0	0
0.99999		7	76	7	0	0	0	0	0	0
1.00000		10	12	0	0	0	0	0	0	0

**Table 8:** Performance comparisons of different algorithms in low dimensional situations.

Type of Obstacles	Algorithms	t(s)	$f_{\mathrm{ave}}$	ψ (%)
Single regular	OABAS	**0.059**	17.63	**100**
	PSO	0.606	19.54	100
	ABC	0.356	17.29	100
Multiple regular	OABAS	**0.083**	17.37	97
	PSO	2.254	19.30	97
	ABC	0.279	17.32	100
Single irregular	OABAS	**0.102**	21.16	**100**
	PSO	7.670	22.68	87
	ABC	0.450	19.29	100
Multiple irregular	OABAS	**0.065**	19.69	**100**
	PSO	17.285	19.52	63
	ABC	0.571	19.90	100
Mixed	OABAS	**0.088**	**19.56**	**100**
	PSO	0.536	21.54	98
	ABC	0.325	21.64	100

**Table 9:** Performance comparisons of different algorithms in high dimensional situations.

Type of Obstacles	Algorithms	t(s)	$f_{\mathrm{ave}}$	ψ (%)
Single regular	OABAS	**0.301**	**36.94**	**100**
	PSO	NA	57.04	52
	ABC	49.729	40.47	96
Multiple regular	OABAS	**0.371**	**41.77**	99
	PSO	NA	51.18	38
	ABC	32.382	48.48	100
Single irregular	OABAS	**0.406**	46.01	99
	PSO	184.593	44.44	39
	ABC	35.434	40.91	100
Multiple irregular	OABAS	**0.428**	41.71	**95**
	PSO	251.506	40.88	12
	ABC	56.813	41.36	48
Mixed	OABAS	**0.345**	**39.91**	**100**
	PSO	NA	57.04	92
	ABC	46.956	48.93	100
